# Transition Shock Experience of Nursing Students in Clinical Practice: A Phenomenological Approach

**DOI:** 10.3390/healthcare10040613

**Published:** 2022-03-25

**Authors:** Yeong-Ju Ko, Soo-Yeon Kim

**Affiliations:** 1 Department of Nursing, Cheju Halla University, Jeju-si 63092, Korea; yjko@chu.ac.kr; 2 Department of Nursing, Deagu-Haany University, Hanuidae-ro 1, Gyeongsan-si 38609, Korea

**Keywords:** clinical practice, nursing student, transition shock

## Abstract

Clinical practice is an irreplaceable educational field in nursing education. Nursing students prepare to become nurses by integrating theoretical and practical nursing practices during clinical practice. However, nursing students experience emotional shock in the clinical environment, which differs from the theoretical learning environment as they enter the clinical practice stage. In this study, the experiences of nursing students with transition shock were analyzed using the analysis method suggested by Colaizzi. From 10 existing subthemes, four new themes were created. Four categories derived from nursing students’ experiences of transition shock were identified as “an unbearable reality”, “feeling the difference between learning and applying nursing care”, “disappointment at the diminished presence”, and “fear of becoming a nurse”. The study results can be used as basic data for measures taken for nursing students in the field of nursing education to increase the understanding of transition shock and its reduction.

## 1. Introduction

Recently, the development of medical technology, changes in the population structure, and healthcare policies required healthcare workers to quickly adapt to changes in the medical field and play a professional role [1]. Nursing educational institutions also aim to train professional nurses to solve health problems through theoretical and practical education [2].

Clinical practice allows students to directly apply the theoretical contents they have learned in school. It is a process in which students can integrate and participate in knowledge and practice [3]. Clinical practice is significant to improving the understanding of the transition to professional nurses [4]. However, many nursing students become nervous because of the unfamiliar hospital environment during clinical practice, feel the difference between the theoretical content learned at school and clinical practice, and experience an immature role performance and lack of confidence due to insufficient knowledge [5,6,7]. In the same context as the transition shock experienced by new nurses, it is necessary to understand the clinical practice of nursing students as a transition process [4].

Transition shock refers to feelings of anxiety, instability, and insufficiency experienced in the roles, responsibilities, relationships, knowledge, and expectations when moving to a new environment [8]. Clinical practice is very important to nursing students. It is necessary to confirm nursing experiences when moving from a familiar environment to a new clinical practice environment for effective practical education. In addition, it is essential to find a way to alleviate and prevent transition shock in the clinical practice environment by confirming the characteristics of the shock.

Previous studies related to the clinical practice of nursing students are mostly quantitative studies that restrictively identify specific variables such as clinical practice stress, clinical practice satisfaction, emotional labor, and self-esteem [9,10,11]. In addition, most of the previous studies related to transition shock were quantitative [5,7], and the transition shock measurement tool used was developed for new nurses [12]. 

However, there are important differences between the roles and responsibilities of nursing students and professional nurses. Existing tools have not taken nursing students’ characteristics into consideration, because their questions presuppose that nurses, as members of the workplace, are licensed to perform medical practices. However, nursing students typically practice observation, and existing tools measure excessive work. Therefore, it is questionable whether previous quantitative studies have accurately measured the transition shock of nursing students.

Therefore, it is necessary to focus on the unique experiences of nursing students’ experiences of transition shock through clinical practice. In addition, it is necessary to understand how to cope with transition shock when changing from a familiar environment to a new clinical practice environment. The purpose of this study is to understand the nature and meaning of transition shock experienced by nursing students in clinical practice and to collect data to develop clinical practice conversion shock tools for nursing students. 

## 2. Methods

### 2.1. Study Design

This study used qualitative research that applied the phenomenological method of Colaizzi [13] to understand the meaning and essence of the transition shock experience of nursing students who have undergone clinical practice.

### 2.2. Participants and Researcher Preparation

#### 2.2.1. Participants

The participants of this study were nine third- and fourth-grade students who had experienced clinical practice at C University in J province and D University in G province. Students with more than two weeks of clinical practice experience who agreed to participate in the study were included as research participants. The participants were students who understood the study purpose and voluntarily agreed to participate in the study. These participants belonged to the researcher’s college, but the study was conducted on students who had no personal interests (such as credit evaluation) in this study.

The research participants were voluntarily recruited through a recruitment notice on the department bulletin board. Nine nursing students who wanted to participate in the study were provided with explanations. After obtaining written consent, an interview was conducted. A sample of nine nursing students were interviewed, an acceptable sample size for a phenomenological study [14]. The sample size was determined by saturation, which refers to the point at which the data collection process fails to yield new information relevant to the study [14].

#### 2.2.2. Researcher Preparation

Researchers had completed the qualitative research methodology in the doctoral program and were experienced in publishing qualitative these. Additionally, researchers had participated in qualitative research conferences, acquired various qualitative research methods, and continued to receive training on qualitative research methods.

### 2.3. Data Collection and Ethical Considerations

Data collection for this study was carried out from 9 June to 2 July 2021, after receiving approval from the Institutional Bioethics Committee of University C (No. 1044348-20210413-HR-001-01).

Data were collected through face-to-face or non-face-to-face (Zoom) personal interviews with researchers. Participants read the recruitment notice on the department bulletin board and were explained the study method and purpose over the phone, which allowed them to participate freely. Before starting the interview, the researchers explained the study purpose, method, and interview recordings. Furthermore, the researchers explained to the study participants that the interview contents would never be used for any purpose other than the research and discarded after the study completion. The researchers explained to the participants the guarantee of anonymity and confidentiality. Besides, the research participants were informed that they could withdraw their participation any time during the research, and no disadvantages would occur even if they discontinued. Written consent was obtained from the study participants before interviews were conducted.

Time, place, and method (Zoom or face-to-face) were recorded during the personal interviews, where participants were free to talk about their experiences. The interviews took approximately 1 to 2 h, and depending on the participants, one to two rounds of interviews were conducted. At the beginning of the interview, the participants were allowed normal conversation. As the interview progressed, semi-structured questions were used. The main question for the interview was, “Tell me about the transition shock you experienced in clinical practice”. Auxiliary questions involved, “How did you feel when you first went to clinical practice?”; “If there is a difference in applying the knowledge and skills you learned in school to clinical practice, please tell me. What emotions/thoughts did you have at that time?”; “Tell me, you know in theory, but were you severely stimulated or shocked by your experience or observation through clinical practice (death/cardiopulmonary resuscitation situation, etc.)?”; “Please tell me if you have experienced or observed severe stimulation, shock, or difficulties in human relationships that you have experienced or observed in clinical practice (nurse-to-nurse, nurse-to-guardian, doctor-nurse, nurse-student).”; “If there is a difference between the role of a practical student you think of and your role as a practical student experienced in clinical practice, please tell me. What emotions/thoughts did you have at that time?”; “If there is a difference between the role and image of the nurse you think of and the role and image of the nurse you experienced in clinical practice, please tell me. What emotions/thoughts did you have at that time?”; “Tell me what kind of effort is needed to reduce and adapt to the traumatic situation experienced during clinical practice (individual/departmental or institutional)?” When confirming the content or clarifying the meaning during the interview, the questions were repeated, and the participants were encouraged to speak. The interview was conducted until theoretical saturation, and data analysis was conducted after the interview.

### 2.4. Data Analysis

The analysis was conducted according to the method suggested by Colaizzi [13]. Coding and qualitative analysis were performed in Microsoft^®^ Word (Microsoft Corp., Redmond, WA, USA) using color highlighting and the “add description” function. Data analysis was conducted in seven steps. First, the researcher read all the interview data several times to grasp its overall meaning, feeling, and the idea of transition shock. Second, important sentences and phrases that contained references to transition shock from individual data were identified. Through this process, two researchers compared the analysis and reached an agreement on its clarity. Third, they formulated meanings from the significant statements through discussion. They then compared the formulated meanings with the original meanings, maintaining the consistency of descriptions. Fourth, the formulated meanings were arranged into clusters of themes, which were aggregated into emergent themes. Following this, the theme-clusters were compared, and a researcher with qualitative research experience checked their accuracy. Fifth, the researchers comprehensively described these terms through their results. Sixth, the researchers attempted to construct a clear statement from the comprehensive description, which could confirm the fundamental theme of the research topic. In the final step, a follow up telephone interview with participants confirmed that the findings reflected their feelings and experiences.

### 2.5. Trustworthiness

The study’s qualitative validity was verified according to the four criteria for qualitative research created by Lincoln and Guba [15]: credibility, transferability, dependability, and neutrality. Credibility was achieved by listening to the recorded content repeatedly, checking it against the transcribed content to ensure its accuracy, and confirming or rectifying any inaccurate or ambiguous parts of it with the participant. It was difficult to know who she was referring to in the interview, and this part was reconfirmed by the participant. The interviewer had the interviewer listen to the transcript again, and it was confirmed that the student explained the situation while listening to the nurse speaking to the colleague. Transferability was ensured by the researcher’s selection of nursing students who had clinical practice experience suitable for the purpose of the study and who could fully express their transition shock. The interview was conducted until theoretical saturation. The researcher sent the results of the study to the participants to confirm whether they matched the participants’ experiences. The results were sent to the subjects through e-mail, and all participants replied that there was no discrepancy. Dependability was achieved through the researcher’s faithfulness to Colaizzi’s analysis method [13] and detailed process description. In addition, during the entire study process, the researcher continued to contemplate the main questions of the study, collecting and analyzing the data. Data dependability was secured through the process of comprehensive review of the manuscript and analysis by researchers with qualitative research experience. To maintain neutrality, prejudice was excluded, and the researcher’s intervention was minimized during interviews, while drawing out the results of the study. Through the process of data collection and analysis, reflections were recorded through an analytical memorandum. To increase result reliability, researchers with qualitative research experience reviewed the interviews to avoid missing content that might be important for the analysis.

## 3. Results

**Figure 1:**
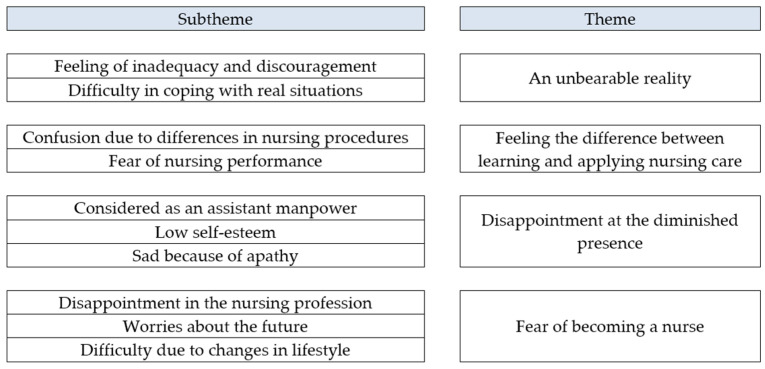
Research findings based on four main themes.

The study recruited nine nursing students, with the average age of 22.55 years, as participants. All of them were females, consisting of five third-grade and four fourth-grade nursing students. The average duration of clinical practice was 8.11 weeks. In this study, 10 subthemes were aggregated to create four themes (Figure 1) as a result of analyzing the nursing students’ experiences on transition shock through Colaizzi’s method [13]. 

### 3.1. Theme 1. An Unbearable Reality

“An unbearable reality” showed a “feeling of inadequacy and discouragement” and “difficulty in coping with real situations”. Unlike the previous classroom practice, the participants were nervous, made mistakes, and realized their limitations when they had to directly nurse or observe the subject in an unfamiliar environment. Additionally, the participants experienced fear and dread about dealing with seriously ill patients and various situations that are difficult to control.

#### 3.1.1. Feeling of Inadequacy and Discouragement

Participants could not ask questions because of fear that they would be reprimanded for their shortcomings. Participants said they made mistakes due to tension in an unfamiliar environment and felt apathy because they realized their limitations.

“*The nurses felt so frightening, and we did not know much about (clinical) work. Whenever we were asked questions, we seemed very cautious. I was afraid the nurse would point out my lack of knowledge. In case you think about the situation, you may not know it either.*”

(ID 6)

“*I think there was a part that made me depressed by talking about what I did not know. From a nurse’s point of view, it was natural for students to know and learn the basics. I was confused as I did not know. I was confused when I asked again, and the nurses said, ‘I am done.’ When the nurse went to pick up (things), it seemed like I made a huge mistake without realizing that I did not know much.*”

(ID 4)

“*The nurse said, ‘Give me this’ in medical terms, but we do not understand it, so what? And when we said we did not know what it was, she changed her words and said, ‘Just get me that,’ and she got angry.*”

(ID 8)

#### 3.1.2. Difficulty in Coping with Real Situations

Participants used simulators in classroom practice. However, in clinical practice, they complained about fear of dealing with situations they directly encountered, such as seriously ill patients, patients’ deaths, and violent situations.

“*(The patient died suddenly) At that time, the family cried a lot. I was worried as if I had become the patient’s family. Contrarily, would I have been able to handle it well if I had been there? I gave it a thought… It’s like, there is nothing I can do about it. For my part (there was nothing I could do)… For the students (there was nothing they could do)…..*”

(ID 3)

“*I was a little scared of seeing a person with the restraint band. The patient shouted ‘let go’ and swore…. I was slightly embarrassed. When I looked at it in a textbook, I accepted it as a written statement. (But) I was terrified of seeing a person with a lack of orientation if I faced the situation. I am a student now, so it’s fine; however, if I were the nurse in charge, how would I control this situation? I think I was worried about this too.*”

(ID 1)

“*In the surgical ward, an older man tried to withdraw an IV (intravenous injection) and refused treatment. I saw him trying to beat the caregiver. The nurse, shorter than the patient, tried to treat him, but he grabbed his wrist and prevented her from touching him. I tried to remove the IV (intravenous). What should I do if (if) it happens to me? No-one tells us. The situation was scary, and I was also afraid to watch it from a distance, so I did not know what to do.*”

(ID 9)

### 3.2. Theme 2. Feeling the Difference between Learning and Applying Nursing Care

In this theme, “confusion due to differences in nursing performance procedures” and a “fear of direct performance” were found. Nursing students directly observed that basic nursing skills in the clinic vary from the theoretical content they learned in the classroom. In clinical practice, unlike the theoretically learned method, basic nursing procedures are simplified and inconsistent between hospitals and nurses, which creates confusion. Additionally, nursing students were concerned that they might harm the patient while performing the technique and were afraid because they lacked confidence.

#### 3.2.1. Confusion Due to Differences in Nursing Procedures

Unlike the content theoretically learned in the classroom, the procedures of basic nursing skills performed in the clinic were simplified. The difference between theory and practice was due to the lack of consistency and various performance methods for each nurse.

“*Even things such as alcohol were different for each nurse. Each hospital has different guidelines. Each hospital uses varied instruments, and the methods used by the nurses slightly differ. Differences between the individuals also exist. Is it correct to do it like that? I thought (heard) this… Therefore, when I go home and look at the (book) again, the order seems irrelevant. What? I am getting a little confused.*”

(ID 2)

“*When I went to the intensive care unit, I saw several acts of aspiration nursing. But (the method) was very different. So I wonder if I should learn the skills again when I am training as a new nurse. But I am a bit confused at this point…*”

(ID 7)

“*I think many people missed it. When I was learning at school, there was an order, but no one seemed to have taken the order into account here. The procedure became very simple after seeing it in action. What do you say? They (skill items) have decreased. There are many procedures when learning at school, but when I went to the hospital, it was very different and simple.*”

(ID 6)

#### 3.2.2. Fear of Nursing Performance

Participants were not confident and were afraid they would make a mistake or be harmed when they had to perform nursing skills directly on the patient.

“*I was checking vitals (vital signs) in the ward or observing. However, there was a huge difference between what I learned theoretically and what I did while talking to the patients in a clinical setting.*”

(ID 9)

“*I went to check the vital signs of the patients and opened the curtains, but all of them were seriously ill. I did not know what to do… I was afraid that I might make a mistake and harm the patient.*”

(ID 2)

“*I have nursing skills for patients… I perform very basic vital measurements or things related to items. I am afraid I might make a mistake.*”

(ID 4)

“*I learned basic nursing, but I was so afraid that (the nurse) might ask me to do some tricks. I was so scared. Really….*”

(ID 6)

### 3.3. Theme 3. Disappointment at the Diminished Presence

In this theme, “considered as an assistant manpower”, “low self-esteem” and “sad because of apathy” were found. Nursing students have the opportunity to apply theoretical knowledge to clinical practice. In the relationship with senior or head nurses, the participants felt a sense of alienation and psychological atrophy as they thought they had no presence. For instance, an assistant worker who was not a subject of education or a folding screen was indifferent and neglected.

#### 3.3.1. Considered as an Assistant Manpower

Participants thought that clinical practice was an opportunity to gain knowledge, skills, attitudes, etc. through direct and indirect experiences in clinical practice as nursing students. However, in actual clinical practice, it was confused with the role of simply assisting nurses’ work.

“*A nurse said, ‘Have a nursing student! Let the student do it!’ This is what the nurse said. Hearing that, we came to learn something… Are we here to help the nurses rather than what we do? A little bit like this….*”

(ID 4)

“*When I thought about practice, I thought I could do what I learned in the clinic. (However) there was only bed making, delivery of pills, and vital sign measurements. Am I here to practice? Or did I arrive as a volunteer or nurse assistant? I thought about it a lot.*”

(ID 7)

“*We went to practice as nurses; however, (in real life) nurses did everything the nurses did, and we felt like assistants or caregivers (roles).*”

(ID 8)

#### 3.3.2. Low Self-Esteem

Participants said they felt like a screen or a statue as they stood still because they did not know where to go during the clinical practice. Their self-esteem was hurt because they seemed to have no presence as nursing students.

“*When I went to the ward, I stood there. The station was too crowded, so (the nurse) told me to stand in the hallway. I stood in the hallway, and if the practice were for eight hours, I would stand for five hours. It may be an exaggeration, but for the past five hours, my legs hurt so much that they became stiff. There were no chairs; I just stood there like the statue or kept going back and forth in the hallway; nobody cared (us). Am I a stone here? I think I am a little angry.*”

(ID 7)

“*My pride is hurt. Am I a folding screen? I felt that way. I was trying to get a blood pressure monitor to measure vital signs, but a nurse got in the way and pushed me. I was hurt when she told me to get out of the way.*”

(ID 4)

“*Many people do not even answer this question. In this situation, we thought a lot, ‘Oh, we are a folding screen.*’…”

(ID 5)

“*The students were inferior to their patients. We felt that way. We will work as nurses in the future. I want you to treat me as a person... as a future nurse.*”

(ID 6)

#### 3.3.3. Sad Because of Apathy

Participants expected to be role models and educators through the appearance of a nurse working in a clinical setting. However, they thought they had neglected their training because of their busy work.

“*We see and learn ourselves. Nurses do not care about us. We must learn independently. The nurses did not take care of us first. When you go to a nurse, the patient’s perineum does not ask to go with you. Don’t worry about us.*”

(ID 3)

“*Nurses did not treat us badly. We practiced for two weeks. As a nurse, I think it might be a little tiring to educate practical students. Still, it plays a role in teaching, but they are not interested in us.*”

(ID 1)

“*The head nurse did not have any training instructions when we went to practice. Do not worry; the practice students change every two weeks.*”

(ID 6)

### 3.4. Theme 4. Fear of Becoming a Nurse

“Fear of becoming a nurse” showed “disappointment in the nursing profession”, “worry about the future”, and “difficulty due to changes in life patterns”. Nursing students can establish career identity and professional intuition through clinical practice. The participants said that they were afraid of becoming a nurse because of the relationship between the nurse’s target and other medical staff and the new nurse’s hospital life, thinking that they would become like other medical professionals in the future.

#### 3.4.1. Disappointment in the Nursing Profession

The nurses observed in clinical practice were not treated as nursing professionals. Participants said they were disappointed when they saw the situation in which a therapeutic and cooperative relationship was not formed between the nurses and other medical staff.

“*The nurses are considered medical professionals. In reality, all medical activities and techniques are performed closely with patients. But I think it is the doctors who get all attention. The nurse feels like a supporting actor behind a flashy light. Will I be able to do so with pride when I later become a nurse? I had that thought. The nurses are doing their best, but I think the nurses are the victims. All nurses are medical professionals. I think the patient will be discharged sooner because of the good cooperation between the medical personnel. However, if the cooperation did not go well, I suppose I would be a little skeptical about that part.*”

(ID 4)

“*It is difficult to work as a nurse in a hospital, but the hospital does not treat you like that. Therefore, I was a little shocked. The nurse went to unload all the specimens….*”

(ID 5)

“*It is different from the theory, but whenever I visit the hospital, I think that the nurses would treat patients kindly. Each patient has its characteristics, but many people treat patients more harshly than expected. Looking at the behavior of the nurses, I felt that it was different from what I had expected…*”

(ID 1)

“*To be honest, looking at patients, I dreamed of becoming a nurse. When I looked at the relationship between the nurse and doctor, who did not get along very well, I did not think I could be a real nurse for a long time.*”

(ID 9)

“*When I look at nurses, they seem a little too much like machines that just work. The nurses seemed too busy. When I went to the hospital, I thought the nurse was like a machine, not a person.*”

(ID 8)

#### 3.4.2. Worries about the Future 

Seeing the rude behavior of senior nurses toward new nurses, the participants said that they were afraid of transforming their future selves and becoming a nurse.

“*It was the first time that a new nurse took over. During the handover, the new nurse was very shaky and paced slowly. The senior nurse next to me was too frustrated to see that, so I pushed the chair and cursed slowly. There were not only nurses but also nursing students. Many eyes were watching. I must have been upset. However, I think this situation can occur at any time. It was my first takeover situation, so I was nervous. I think it is about learning and growing. I do not know what happened before, but based on the circumstances, will my future be like that? It must be a little difficult… Maybe it is my future self…..*”

(ID 9)

“*Before the practice, I thought that I would endure it to a certain extent, even if there was a bully. I thought the image of the nurse was an angel. After practicing, I understand that the nurses are strict and busy. However, after experiencing it, I learned what killing people with words felt like.*”

(ID 5)

“*Maybe it is necessary to talk about what the new nurse did wrong and make sure you do not make that mistake again. I do not know if the discipline should end. However, I think it is wrong for a senior nurse to gossip about a new nurse in front of others. I could be a target in the future.*”

(ID 8)

#### 3.4.3. Difficulty Due to Changes in Lifestyle

The participants continued to practice in a tense state during clinical practice because of an unfamiliar environment, had to work in shifts according to the practice schedule, and experienced physical and mental fatigue because of parallel practices and tasks.

“*Going to the hospital is stressful. My shoulder was so stiff that when I went to the (hospital), my body hurt so much…. I was too nervous.*”

(ID 2)

“*Waking up (early) in the morning was a bit tiring. When I return home after finishing (practice) at around 3 pm, the time passes so quickly that I directly go to sleep. And in the morning, I get up and go to the hospital… Life is repetitive. Therefore, I think it is more difficult. Even while visiting the university, I did not have to use my physical strength more, as I sat for long hours. (However) During practice, I spent more time standing. I did more active things. Therefore, it becomes physically arduous. I think it was difficult because we had to keep doing the assignments.*”

(ID 1)

“*It was so hard. All students in the clinical practice said the same thing. Day shifts are difficult (getting up in the morning), but I can rest in the afternoon (remaining) after work. So, I complete the rest of my studies in the afternoon. However, in the evening, I become so tired that I keep on sleeping. When I get home in the evening, it is around 10 or 11 pm, and the day is over.*”

(ID 6)

## 4. Discussion

This study attempted to understand the meaning and essence of the transition shock experienced by nursing students during clinical practice when they changed to a new clinical practice environment and derived four categories as a result.

The first theme was “an unbearable reality”. Unlike the familiar classroom practice environment, the participants did not ask whether they would be rebuked for their lack of knowledge in the new clinical environment. The participants felt their limitations and withdrew. They experienced fear of dealing with the death or severe patients they encountered for the first time. Kim, Sun, and Kim [16] found that second-year nursing students are unfamiliar with classroom practice, which is familiar and controllable in introductory clinical practice. Additionally, the practice environment is unfamiliar and has a heavy burden, making it difficult and stressful, similar to the study result. In a study by Kim and Song [17], even nursing students who completed introductory clinical practice felt inadequate because they did not learn all the theoretical courses when they started practice. Furthermore, the results are similar to those of Kang and Choi’s study [18], showing that nursing students fear the sudden death of a patient during practice, and a sense of helplessness arises that they could not do anything. Therefore, for nursing students to reduce anxiety and tension in unfamiliar clinical settings and engage in practice with confidence, it is necessary to develop and improve various educational methods enhancing the clinical practice presence in classrooms. Furthermore, when experiencing difficult situations, such as death, it is necessary to have a preparation time to think about the role that a nursing student can play in advance to have coping skills.

Regarding the second theme, “feeling the difference between learning and applying nursing care”, the participants felt confused because the basic nursing procedure provided to patients in the clinic varied from what they learned in the classroom. Participants said that they lacked confidence performing nursing on the subject and were worried that it would harm them. In a study by Kim et al. [16], nursing students experienced a difference between what they practiced in the clinical setting and classroom. The procedures and standards of nursing skills practiced in the clinic are different from what they have learned theoretically. It can be seen that the study results were similar to those of confusion. In a study by Sin, Kwon, and Kim [19], of the contents of reality shock perceived by new nurses, concerns about harming the patients were the highest. In a study by Sin and Kim [20], new nurses encountered embarrassment as they experienced that the knowledge they learned at school varied from practice. Besides, senior nurses showed a lack of professionalism by not following principles when performing nursing, which is consistent with the results. Nursing students can gain confidence and a sense of accomplishment by applying what they learn in school to actual subjects through clinical practice. To achieve such a positive effect, preparation of theory and practice is necessary [5]. Therefore, to confidently perform nursing behavior in actual subjects, it is essential to prepare for the theory and technique before the practice. There is a need to develop continuous measures to reduce the gap between clinical and theoretical practice.

The third theme is “disappointment at the diminished presence”. The participants thought it to be an opportunity to apply or expand the knowledge they had theoretically learned through clinical practice. In actual clinical practice, the role of nursing students was either a nursing assistant or they did not seem to have a presence. In a study by Yang et al. [21], during clinical practice, nursing students perceived their existence as invisible human beings and showed atrocious and timid appearances. Cho and Chun [22] stated that they felt helpless because they could not do anything because of the limited practice focused on observation. Furthermore, in the study by Song and Kim [23], for developing clinical practice education, it is necessary to secure a qualified practical instructor as an improvement item for the institution and to increase the pride and responsibility for the education. To reduce the feeling of helplessness experienced during practice, it is necessary to clearly define and standardize the roles and standards of nursing students through continuous consultation with the practice organization [17]. Therefore, to apply to nursing students’ practice or expand their knowledge through clinical practice, it is necessary to have a systematic plan to increase the specific educational guidelines and sense of responsibility of field leaders through consultation with practice organizations. Additionally, it is necessary to clarify the roles and standards of nursing students in practice.

The fourth theme was “fear of becoming a nurse”. It was a disappointment to see that nurses were not respected as professional nurses in the clinical field. It means that the participants were transferred to their future selves as they watched new nurses work and experienced difficulties adapting to shift work. In a study by Kim and Song [17], nursing students said they thought about the future while examining themselves through the appearance of new nurses they met in clinical practice. The participants feel disappointed for not being treated as a professional in the actual work of new nurses and felt confused about their values [20]. It is said that the practice students develop nursing professionalism and establish their identity through the image of a nurse, demonstrating competency as a professional nurse observed in clinical practice [24]. Therefore, nursing students must sufficiently think about the nursing profession. Institutions also need to generate a plan to form proper nursing professionalism, such as creating a culture that can establish the image of nurses as professional nurses and managing continuous respect among nurses. 

This study describes the transition shock experienced by nursing students at the stage prior to their adaption to practice and based on this, suggests coping strategies to adapt to clinical practice. Students’ pressure at having to treat patients in a real-life situation which is different from practice at educational institutions made the acceptance of reality difficult. The burden of reality demands ways of overcoming it in a dimension different from simulation or virtual reality programs. Furthermore, students become suspicious of the differences between what they learn and what they apply. In a clinical setting, when there is a conflict between protocols or when priority must be given in a patient situation, nursing skills applicable to multiple situations should be taught. In addition, it is necessary to introduce a mentoring program so that students can feel a sense of belonging to their ward during the practice period.

## 5. Conclusions

This study attempted to understand the meaning and essence of nursing students’ experiences of clinical practice transition shock through a phenomenological research method. The study result shows that nursing students felt an unbearable reality, the difference between theory and practice, and sad that they were not recognized as practical students and experienced fear of becoming a nurse.

Nursing students’ clinical practice is an opportunity to apply what they have learned in theory to practice, as it is significant to learn the skills and abilities required in nursing practice. Nursing students can establish professionalism and identify themselves as future nurses through clinical practice courses. Therefore, to increase the effectiveness of practical education, an effective strategy is required for the operating system, methods, and content of practical education. Universities need to generate an improvement plan through continuous consultation with the practice institution. In addition, to reduce the transition shock, it is necessary to develop a tool that can measure the transition, including the contents.
